# Tracking the fate of iron in early development of human blood flukes

**DOI:** 10.1016/j.biocel.2007.04.017

**Published:** 2007

**Authors:** Malcolm K. Jones, Donald P. McManus, Padma Sivadorai, Amber Glanfield, Luke Moertel, Sabina I. Belli, Geoffrey N. Gobert

**Affiliations:** a Queensland Institute of Medical Research, Herston, Qld 4029, Australia; b School of Veterinary Sciences, The University of Queensland, Brisbane, Qld 4072, Australia; c School of Population Health, The University of Queensland, Brisbane, Qld 4072, Australia; d Institute for the Biotechnology of Infectious Diseases, University of Technology Sydney, Broadway, NSW 2007, Australia

**Keywords:** Cu, copper, DOPA, dihydroxyphenylalanine, EM, electron microscopy, Fe, iron, Fer1, ferritin 1, Fer2, ferritin 2, ICP-MS, inductively coupled plasma-mass spectroscopy, LMM, laser microdissection microscopy, RT-PCR, reverse transcriptase polymerase chain reaction, TEM, transmission electron microscopy, *Schistosoma japonicum*, Schistosomiasis, Ferritin, Eggs, Iron, Quinone tanning

## Abstract

Iron (Fe) is an important trace element found in nearly all organisms, and is used as a cofactor in many biological reactions. One role for Fe in some invertebrates is in stabilization of extracellular matrices. The human blood fluke, *Schistosoma japonicum*, is responsible for significant human disease in developing and tropical nations. Disease in humans arises from host immunological reaction to parasite eggs that lodge in tissues. Schistosomes require Fe for development in their hosts, and store abundant Fe in vitelline (eggshell-forming) cells of the female system. The understanding of Fe metabolism and functionality are aspects of its biology that may be exploited in future therapeutics. The biology of Fe stores in vitelline cells of *S. japonicum* was investigated to illuminate possible functions of this element in early development of these parasites. Vitelline Fe is stored in yolk ferritin that is upregulated in females and is also expressed at low levels in egg-stages and adult males. Laser microdissection microscopy, coupled with reverse transcriptase- and real time-PCR amplification of schistosome ferritin sequences, confirmed that the vitelline cells are the likely progenitor cells of yolk ferritin. Assessment of Fe concentrations in whole male and whole female adult worms, eggs and purified eggshells by colorimetric assays and mass spectroscopy demonstrated higher levels of Fe in the female parasite, but also high levels of the element in whole parasite eggs and purified eggshell. Qualitative energy dispersive spectroscopy of purified eggshells, revealed that Fe is abundant in the eggshell, the matrix of which is composed of heavily cross-linked eggshell precursor proteins. Thus, vitelline stores of Fe are implicated in eggshell cross-linking in platyhelminths. These observations emphasise the importance of Fe in schistosome metabolism and egg formation and suggest new avenues for disruption of egg formation in these pathogenic parasites.

## 1 Introduction

Iron (Fe) is an essential factor in a wide range of redox reactions that occur in nearly all organisms. Fe-containing proteins catalyse vital reactions associated with oxygen and electron transport, energy transduction, folate metabolism, nucleic acid synthesis and detoxification. Parasitic protistans and metazoans are as dependent on Fe as related free-living taxa, although some parasite groups may have lost pathways for Fe acquisition as the parasitic life-style was adopted (Rao, Carta, Lesuisse, & Hamza, 2005). The malaria parasites require Fe for intracellular proliferation. The selective intracellular chelation of Fe can stop growth of these parasites within erythrocytes (Walcourt, Loyevsky, Lovejoy, Gordeuk, & Richardson, 2004; Wilson & Britigan, 1998).

Schistosomes are metazoan parasites responsible for significant human morbidity and mortality in tropical and developing nations (King, Dickman, & Tisch, 2005; Ross et al., 2002). The adult parasites inhabit veins of the mesenteries and vesicle plexus, where they ingest erythrocytes as a source of amino acids and absorb a range of low molecular weight solutes across their body surface (Jones, Gobert, Zhang, Sunderland, & McManus, 2004; Jones, Randall, McManus, & Engwerda, 2004). This intravascular environment provides adult parasites with high levels of serum Fe, carried either as a component of heme, or in the serum transporter transferrin. However, little is known about the molecular mechanisms that allow these parasites to use this rich resource of Fe.

It appears that other haematophagous metazoans have significant requirements for both elemental Fe and Fe-compounds, such as heme (Graca-Souza et al., 2006). Schistosomes are dependent on host Fe for early development within the mammalian host (Clemens & Basch, 1989) and females are particularly enriched for this element (WoldeMussie & Bennett, 1982). Fe appears to be absorbed across the surface tegument of the parasites, possibly by stripping host transferrin of its metabolic cargo (Clemens & Basch, 1989). Subsequent transport of divalent Fe may be mediated by members of a family of divalent metal transporters, present in the schistosome tegument (Smyth et al., 2006).

An interesting aspect of Fe metabolism in schistosomes relates to the distribution of ferritin isoforms in the adult worms. Two isoforms of this intracellular iron storage molecule are expressed in adult *S. mansoni*. The first isoform, the so-called schistosome yolk ferritin (Fer1) is expressed preferentially in females, as measured at transcript and protein level in *S. mansoni*. The second isoform, somal ferritin (Fer2), is preferentially expressed in males (Dietzel, Hirzmann, Preis, Symmons, & Kunz, 1992; Schüssler, Potters, Winnen, Bottke, & Kunz, 1995; Schüssler et al., 1996). Schistosome yolk ferritin is thought, on the basis of electron microscopy studies, to be present in the yolk platelets of vitelline cells, thereby contributing, as a component of yolk, to the developmental requirements of the developing miracidia (Schüssler et al., 1995).

Vitelline cell of adult schistosomes and related taxa is a specialized accessory cell of the female reproductive system. Vitelline glands (or follicles) liberate mature vitelline cells as entire cells. At the time of release from the gland, the cells are replete with eggshell precursors bound in secretory vesicles. In the egg-forming complex of the female system, numerous vitelline cells surround a single fertilized ovum, and upon appropriate signal, release the eggshell precursors into the lumen of the egg mould complex. Under action of tyrosinases and other enzymes phenol oxidases, the abundant tyrosine residues of the precursor proteins are oxidised to dihydroxyphenylalanine (DOPA) and quinone (Ebersberger, Knobloch, & Kunz, 2005; Fitzpatrick, Hirai, Hirai, & Hoffmann, 2007; Smyth & Clegg, 1959). The primary role of the vitelline cells, therefore, is choriogenesis. It is less certain that the vitelline cells have a true yolk function in nourishing the developing embryos. Certainly, spent vitelline cells are entrapped with the zygote within the polymerised eggshell. The absence of their cellular remains in the fully mature egg implies that their cellular components are absorbed by the embryo. Whether any of the secreted components of the vitelline cells are incorporated by the embryo is not known. For schistosomes, there is some evidence that the intra-ovular embryo accesses nutritional source from the host environment (Ashton, Harrop, Shah, & Wilson, 2001). Schüssler et al. (1995) proposed two possible functions for yolk ferritin in schistosomes, namely, as a source of Fe for larval development, or as a means of combating bacterial infection in the larva after it is released from the human host. To date there is no information on processing of the Fe in embryogenesis.

Quinone tanning reactions are used by many invertebrate groups to produce strongly cross-linked proteins. Fe plays an important role in cross-linking and stabilization of DOPA-rich proteins of mussels (Sun & Waite, 2005; Yamamoto et al., 1990). There are no data demonstrating Fe in the stabilization of eggshell precursors during egg formation in platyhelminths, due in large part to the inherent difficulties in working with the eggs of these worms (Ebersberger et al., 2005).

This paper investigates two related issues concerning the biology of Fe-stores in the vitelline cells of schistosomes. The first issue concerns the cellular origins of ferritin proteins that are present in vitelline cells. Although Schüssler et al. (1995) demonstrated by morphological EM the presence of ferritin particles in mature vitelline cells, they stated that it was probable that ferritin was transferred to vitelline cells by progenitor cells. The second issue addresses the embryonic fate of Fe-stores in vitelline cells. In this we ask specifically whether Fe is incorporated into the eggshell of the embryo. From these studies, we present a model of Fe biology in early development of schistosomes, with consequent insight into the biology of the complex and enigmatic vitelline cells of platyhelminth parasites.

## 2 Materials and methods

### 2.1 Parasites

*Oncomelania hupensis hupensis* snails, infected with Chinese (Anhui) *Schistosoma japonicum*, were obtained the Institute of Parasitic Diseases, Shanghai, China. Adult worms were perfused 6 weeks after challenge from ARC Swiss mice infected percutaneously with 40 cercariae shed from snails. Schistosome eggs were obtained from mouse liver by digestion with collagenase B (Dalton, Day, Drew, & Brindley, 1997), or were purified from faeces and intestinal content of necropsied mice by passage through copper mesh and centrifugation.

### 2.2 Stereological analysis of vitelline regions

The relative abundance of vitelline cells in vitelline-enriched regions of females was studied by transmission electron microscopy and point-counting stereology (Griffiths, 1993). Parasites were fixed in 3% glutaraldehyde in phosphate buffer, followed by 1% osmium tetroxide and embedded in Epon resin. Ultrathin sections of females were examined by JEM1011 transmission electron microscope operated at 80 kV. Point-counting stereology was used to estimate the volume of tissue occupied by vitelline cells within the vitellogenic regions of the parasites. Random sections of vitellogenic regions of females were selected and five photographs were taken from non-overlapping and non-adjacent regions, following methods described previously (Bartley et al., 2006). Grids generated by the ImageJ analysis software (NIH, Bethesda) were laid over each image for point counting. Volume density of vitelline cells was estimated from the number of grid points intersecting a cell divided by the numbers of points intersecting all tissues in the vitellogenic region.

### 2.3 Analysis of ferritin expression in specific schistosome tissues

Fresh adult parasites were embedded in OCT embedding compound and snap-frozen on dry ice. Frozen blocks were stored at −80 °C until sectioned. Sections (6–10 μm thick) were cut by cryostat using sterile knife blades onto polyethylene naphthalene (PEN) membrane coated glass slides (P.A.L.M. Microlaser Technologies, Bernried, Germany). Slides were stored at −80 °C. Slides were washed in diethylpyrocarbonate (DEPC)-treated water to remove OCT, stained with 1% Mayer's haematoxylin or with 1% methyl green and refrozen until further use. Before use, slides were allowed to thaw under sterile conditions for approximately 15 min. Vitelline material and control tissues (from parenchymal cells from male worms and female non-germinal tissues) were microdissected using a PALM MicroBeam Laser Catapult Microscope (P.A.L.M., above).

In one set of experiments, expression of ferritin genes in vitelline and control tissues was analysed by reverse-transcriptase PCR. Total RNA from laser microdissection microscopy (LMM)-isolated cells was extracted and purified using TRIzol reagent (Invitrogen). In addition, whole adult worm was also processed in TRIzol for RNA isolation. Quantitative analysis of the RNA from vitelline and control samples was performed using a ND-1000 Spectrophotometer (Nano Drop, Wilmington, USA).

A One-Step RT-PCR kit (QIAGEN) was used and the protocol followed according to the manufacturer's instructions. Primer pairs were designed against the two *S. japonicum* ferritins (GenBank accessions given earlier) and schistosome triphosphate isomerase (TPI) (see Oliveira, Busek, & Correa-Oliveira, 1998). For these experiments the primers sets amplified transcripts of approximately 500 bp (Table 1). One round of PCR was performed following the manufacturers instructions, with a PCR program as follows: 50 °C for 30 min, 95 °C for 15 min, and then 40 cycles of 94 °C for 30 s, 50 °C for 30 s, 72 °C for 1 min, and a final 72 °C for 10 min. The RT-PCR products were run onto a 1% (w/v) agarose gel and detected with ethidium bromide.

Total RNA from laser microdissection microscopy (LMM)-isolated cells was extracted and purified by the standard QIAGEN RNeasy mini kit protocol. In addition, separate male and female adult parasites were also processed for total RNA isolation using TRIzol reagent. Quantitative analysis of the RNA from vitelline and control samples was performed using a ND-1000 Spectrophotometer. Complementary DNA was synthesised by use of a SuperScript III Platinum CellsDirect Two-Step qRT-PCR kit (Invitrogen, Mount Waverley, Australia) using vitelline and control cells from female *S. japonicum* adult worms. The cDNA samples were quantified by a ND-1000 spectrophotometer and stored at −20 °C.

For real time-PCR, a master mix was made according to CellsDirect protocol (Invitrogen) and combined together with 4 μg of samples in individual 0.1 ml tubes (Corbett Research, Sydney, Australia). Primers sets for amplification of *S. japonicum* yolk ferritin (ferritin 1) and somal ferritin (ferritin 2) are shown (Table 1). All reactions were performed on a Rotor-Gene (3000) real time-PCR (Corbett Research) and analysed by Rotor Gene 6000 Software version 1.7 (Corbett Research). Real time-PCR parameters were set by determination of primer melting temperature and addition of a melt curve to show primer viability. Complementary DNA from whole adult *S. japonicum* worms, together with primers for the two *S. japonicum* ferritins (Table 1), was used as a positive control and standard curve for all real time-PCR experiments.

### 2.4 Stage and gender specific expression of ferritins in *S. japonicum*

Total RNA was isolated from single sex adult schistosomes (100–200) or eggs using published protocols (Hoffmann, Johnston, & Dunne, 2002). The visualisation of total RNA quality/quantity was assessed using the Bioanalyzer RNA Nano LabChip (Bioanalyzer) (Agilent, Santa Clara, USA) as described (Moertel et al., 2006). Complementary DNA was synthesised from total RNA from eggs and adult mixed sex parasites using a modified SuperScript™ III protocol (Invitrogen, Melbourne, Australia) with random hexamers (Invitrogen). The primer sets for each amplicon are presented in Table 1. All cDNA samples were diluted to 100 ng/μl, quantified by a ND-1000 spectrophotometer (Nano Drop, Wilmington, USA) and then 5 μl aliquots were combined with 10 μl of SYBER^®^ Green (Applied Biosystems, Foster City, USA), 3 μl of water (Sigma) and 2 μl (5 pmol) of the forward and reverse primers in a 0.1 ml tube (Corbett Research, Sydney, Australia). All reactions were performed as described above. Real time PCR parameters were set by determination of primer melting temperature and addition of a melt curve to show primer viability. *Schistosoma japonicum* glyceraldehyde-3-phosphate dehydrogenase (GAPDH) was used as a positive control and standard curve for real time PCR experiments (Moertel et al., 2006).

### 2.5 Assessment of iron content in schistosome tissues

#### 2.5.1 Colorimetric assay for iron

Concentrations of non-heme iron in adult males, females and eggs of *S. japonicum* were determined colorimetrically using a modification of the method of Torrance and Bothwell (1968). This procedure was performed in duplicate. Parasite samples were dried by baking at 80 °C for 48 h. The dried samples were then weighed and digested in 150 μl of concentrated nitric acid at 250 °C for 3 h. Aliquots (100 μl) of mineralised samples were transferred into new plastic tubes, to which 5ml of working chromogen reagent (one volume of aqueous 0.1% (w/v) aqueous bathophenanthroline sulphate and 1% (w/v) thioglycollic acid mixed with five volumes of water and five volumes of saturated sodium acetate) was added. A standard iron solution (Sigma–Aldrich Inc.) diluted to 2 mg/ml in 0.5% HCl (Aristar) and a negative control (50% HNO_3_ and 50% distilled H_2_O) were prepared. Iron concentrations were determined spectrophotometrically at an absorbance of 535 nm, and calculated using the standard formula in Torrance & Bothwell (1968).

#### 2.5.2 Inductively coupled plasma-mass spectroscopy (ICP-MS)

ICP-MS was also used to determine iron and copper concentrations in livers from uninfected mice, livers from *S. japonicum*-infected mice, intact eggs (which contained miracidia in various stages of development), and purified eggshells. The eggshells were obtained from eggs that had been purified from livers by collagenase B digestion. These eggs were cracked open by a homogenizer equipped with a plastic spin bar. Homogenization was conducted for four cycles of 30 s duration over an ice bath to minimise temperature increases in the samples. Samples were examined by brightfield microscopy to ensure that the eggshells has ruptured. The eggshells were subsequently incubated in 2% aqueous potassium hydroxide (KOH) for 2 h at 37 °C to remove soft tissues. After incubation, eggshells were re-examined microscopically to ensure that all soft tissues had been removed. The purified shell material was washed in repeated changes of distilled water to remove excess KOH.

Samples were mineralised in high purity concentrated nitric acid and dried to obtain dry weight. The samples were then resuspended in nitric acid and incubated at 90 °C for 1 h to ensure complete dissolution of the samples. Samples were cooled and diluted to volume with deionised water. The diluted digests were analysed for concentrations of Fe and copper on a Varian Vista Pro Inductively Coupled Plasma Optical Emission Spectrometer.

### 2.6 Energy dispersive spectroscopy

*S. japonicum* eggs, obtained from collagenase digestion of livers or purified from faeces, were transferred from PBS into distilled water to facilitate hatching. Schistosome eggs hatch spontaneously in freshwater (Kusel, 1970) and for *S. japonicum*, many larvae have emerged after 1 h in freshwater. When *S. japonicum* hatches, the entire contents of the shell, including the vitelline membrane, are disgorged (Jones, Glanfield and Sze Bong, unpublished observations) and clean, uncontaminated shell remains. Eggs shells from hatched eggs were transferred by pipette into fresh distilled water and mounted onto formvar-carbon coated grids for electron microscopy and energy dispersive (X-ray) spectroscopy (EDS), which was performed in an FEI Tecnai F20 field emission gun (FEG) transmission electron microscope operated at 200 kV. The microscope was operated at a low FEG emission current of 3800 V in scanning transmission electron microscopy (STEM) mode with the condenser aperture 1 set to 400 μm, condenser aperture 2 set to 100 μm and a nanoprobe size set to 9. These settings produce a probe that is approximately 3 nm in diameter. Both bright field (BF) and dark field (DF) images were obtained in scanning transmission electron microscopy (STEM) mode configuration with the sample tilted at 25° towards the EDS detector. Count rates of up to a few hundred counts per second were acquired for the EDS spectra for 60 live seconds.

## 3 Results

### 3.1 Ultrastructural and stereological analysis of vitellogenic regions of females

**Fig. 1:**
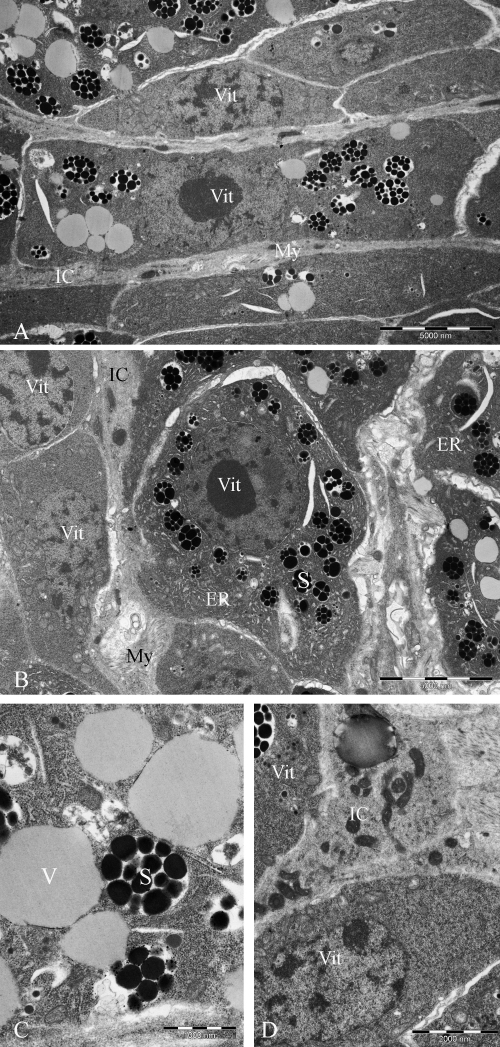
Vitelline tissue of *S. japonicum*, transmission electron microscopy. (A) Vitelline cells within a follicle. The cytoplasm of the vitelline cells is moderately electron opaque and distinguishable from the lucent cytoplasm of interstitial material. Myofibrils are occasionally observed among the interstitial cells, indicating the presence of cells of the musculo-parenchymal system. (B) A mature vitelline cell, with abundant shell globules. Note the presence of interstitial cells with myofibrils. (C) Vitelline cell. Shell globules flanked by yolk platelets. (D) Interstitial cell in vitellogenic region. *Abbreviations*: IC: interstitial cell; ER: endoplasmic reticulum; My: myofibril; S: eggshell precursor globule; V: yolk platelet; Vit: vitelline cell.

The vitelline follicles of *S. japonicum* form a series of compact, multicellular glands in the posterior two-thirds of the female worms. In these regions, the glands surround the voluminous gut and are surrounded, in turn, by a thin parenchymal layer, transverse and longitudinal muscles bundles and tegument. A thin cellular layer of musculo-parenchymal tissues occupies the layers between vitelline follicles and gastrodermis. Each mature vitelline cell (Fig. 1A and B) has a large moderately electron opaque cytoplasm surrounding a central nucleus. Rough endoplasmic reticulum and Golgi bodies abound in the cytoplasm, as do two forms of vesicular inclusion body, the shell-precursor vesicles and yolk platelets (Fig. 1A and D) (see Schüssler et al., 1995). The shell precursors were evident as large bodies in which occurred numerous electron-opaque shell globules (Fig. 1C). Electron-lucent yolk platelets have a homogenous, lucent cytoplasm and possess small particles, interpreted by those authors to be ferritin (Schüssler et al., 1995), in the matrix. Immature vitelline cells are smaller, but possess a cytoplasm of similar electron opacity. Interstitial cells lying among vitelline cells and follicles are elongate cells, with a generally electron lucent cytoplasm that is otherwise unremarkable (Fig. 1A, B and D). Occasionally, thin myofibrils (Fig. 1A and B) are seen among the interstitial material, indicating that some of the cellular material in the intervening layers is musculo-parenchymal tissue.

Stereological analysis of volume densities of cells in vitellogenic regions showed that despite the presence of these intervening cells, the vitelline regions still occupied 81.6% of tissue volume; whereas other cells occupied 12.2% and the extracellular matrices approximately 6.2% of tissue volume. Therefore, the proportional volume of cellular material occupied by vitelline cells was 83.7% of total cell volume in vitellogenic regions.

### 3.2 Stage and gender-specific expression of schistosome ferritins

**Fig. 2:**
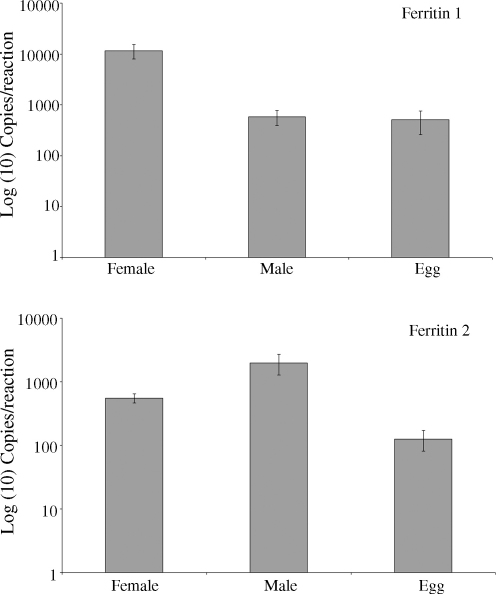
Quantitative analysis of stage- and gender-specific expression of schistosome yolk ferritin (ferritin 1) and somal ferritin (ferritin 2) in *S. japonicum*, by real-time PCR. The data are derived from four replicates.

Real-time PCR analysis of expression of ferritins in *S. japonicum* male and female worm extracts and eggs revealed that female worms express abundant yolk ferritin (Fer1) transcripts (Fig. 2) at levels approximately 15 times that of eggs and males. Levels of somal ferritin (Fer2) transcripts were approximately 4 times higher in males than females and 16 times higher in males than embryos (Fig. 2).

### 3.3 PCR amplification of iron-regulating genes

**Fig. 3:**
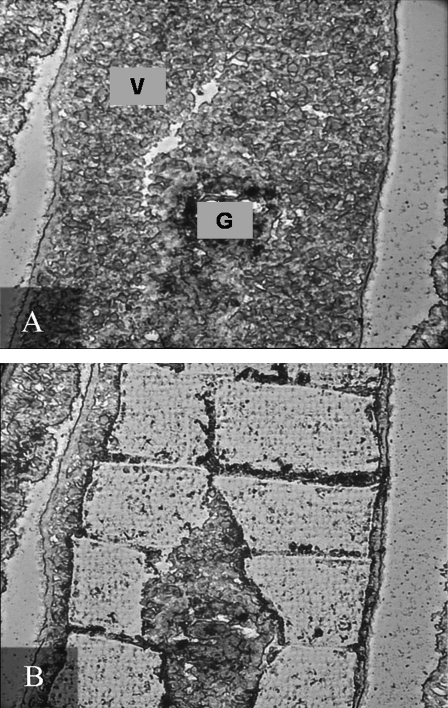
Unfixed cryostat section of female *S. japonicum*, stained with methyl green, before (A) and after (B) laser microdissection microscopy. *Abbreviations*—V: vitelline cells; G: gut.

Vitellogenic regions were laser microdissected as a source of vitelline cell-enriched messenger RNA (mRNA). A sample section of an unfixed female parasite is shown before and after laser microdissection (Fig. 3). Vitelline cells (V) were easily identifiable by their bright blue/green appearance in female worms after staining with methyl green. Approximately 900,000 μm^2^ of vitelline enriched material tissue and 700,000 μm^2^ of control tissue were microdissected. After RNA purification, spectrophotometric analysis of samples revealed a yield of 7.5 ng/μl RNA (*A*_260_/*A*_280_ of 1.72) for vitelline enriched extracts and 6.1 ng/μl RNA (*A*_260_/*A*_280_ of 1.75) for control extracts.

**Fig. 4:**
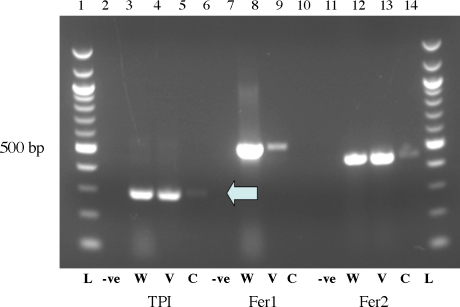
Gel electrophoresis of *S. japonicum* yolk ferritin (Fer1), somal ferritin (Fer2) and TPI. Total RNA was obtained from whole adult worms (W), and laser dissected control tissue (C) and vitelline tissue (V). TPI was used as a positive control as the molecule is expressed in all tissues. Lanes marked with a minus sign (−) are negative controls, for which no RNA template was added to RT-PCR reactions. TPI and Fer2 was amplified in all three tissue samples, while Fer1 was observed in whole worm and vitelline tissues after one round of PCR. *Abbreviation*: L: 100 bp molecular weight ladder.

Aliquots from microdissected tissues and control whole worm tissues were subjected to RT-PCR analysis using primer sets for the constitutively expressed TPI and the isoforms of ferritin (Fig. 4). TPI was expressed in all tissues (Fig. 4, lanes TPI-W, TPI-V and TPI-C), although the weakest band was present in non-vitelline tissues. For yolk ferritin, bands were present in whole worm extracts and vitelline cell extracts (Fig. 4, lanes Fer1-W and V), but not in control tissues (lane Fer1-C), whereas somal ferritin (Fig. 4, Fer2, lanes W, C and C) was present in all lanes and appeared to be expressed in all tissues represented in the extracted samples.

In addition, total RNA from microdissected tissues were subjected to real time PCR analysis using primer sets for the isoforms of ferritin. Approximately 900,000 μm^2^ of control and vitelline-enriched tissue was microdissected. Real time PCR was employed to quantify the abundance of ferritin 1 and ferritin 2 in LMM isolated vitelline and parenchymal tissues of the adult female *S. japonicum*. Relative abundance of yolk ferritin (ferritin 1) in vitelline cells was 1067.8 ± 110.8 (*n* = 7) while in control tissues it was 93.7 ± 44.7 (*n* = 8), indicating an 11.4-fold relative higher abundance in the vitelline-enriched extract. The abundance of somal ferritin gave mixed results and did not appear differentially expressed in either RNA population. However, to confirm that transcripts for somal ferritin were present in material prepared for real time PCR, we scraped sections from slides using a sterile razor blade, extracted RNA and subjected this to RT-PCR. A weak band at the predicted size was observed (data not shown).

### 3.4 Assessment of elemental iron and copper content in schistosome eggs and eggshells

#### 3.4.1 Colorimetric assay and inductively coupled plasma-mass spectroscopy

**Fig. 5:**
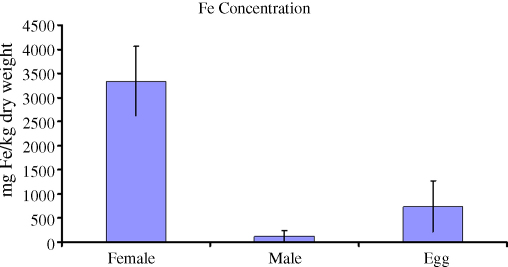
Colorimetric assay of Fe concentrations in schistosome males, females and eggs of *S. japonicum*, expressed as μmol/g dry weight.

All samples were mineralised in concentrated nitric acid solutions and comparative non-heme iron concentrations in the tissues of *S. japonicum* adults and eggs are shown (Fig. 5). Iron concentration in females was greater than the corresponding value in eggs, which in turn were greater than males. Fe and copper concentrations determined from ICP-MS analysis of host tissue, infected liver, intact eggs (eggshell plus enclosed miracidium) and purified eggshells are shown (Table 2).

### 3.5 Energy dispersive spectroscopy

**Fig. 6:**
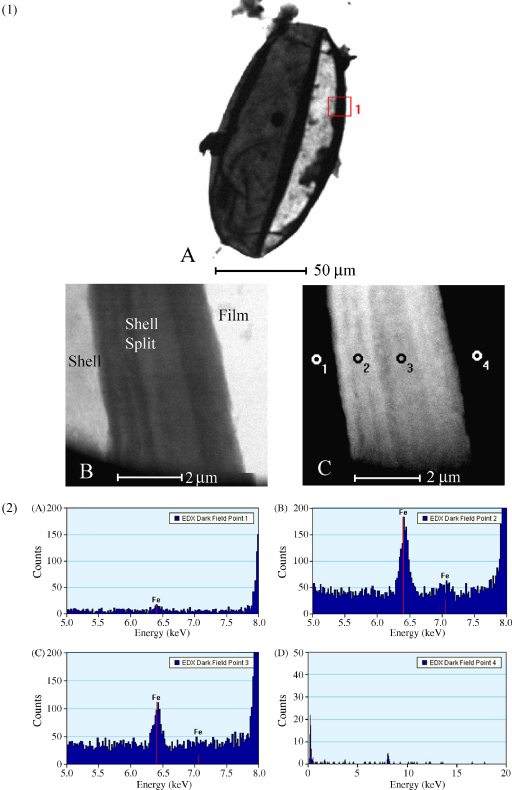
EDS of *S. japonicum* eggs purified from mouse faeces. Panel (1) (left). Images of: (A) whole egg; (B) enlargement of boxed region of Panel 1A, bright field. (C) Enlargement of boxed region of Panel 1A, dark field. The four points indicate (o_1_–o_4_) points where EDS spot analyses were performed. Panel 2. Representative EDS spectra of spot analyses obtained from the four points indicated on Panel 1C. Point 1 lies over a single layer of eggshell. Points 2 and 3 lie over the hatching-induced longitudinal split in the shell. The shell rolls slightly at this point, so that there are multiple layers of shell, thereby increasing the abundance of egg tissue in the beam path. Point 4 lies over the carbon film immediately next to the egg.

**Fig. 7:**
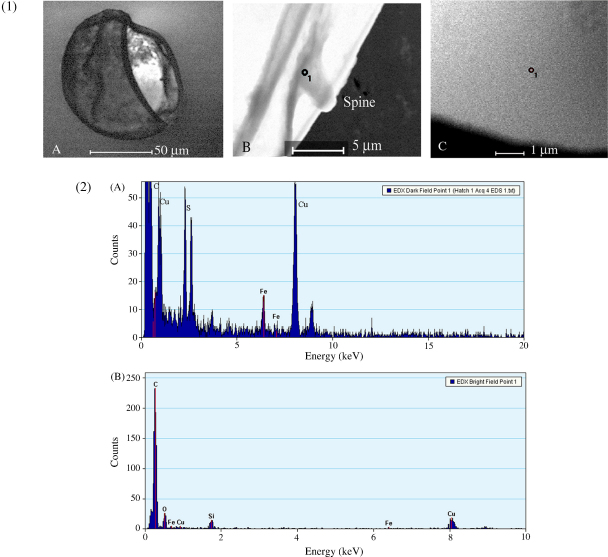
EDS of *S. japonicum* eggs purified from liver and of the formvar-carbon film. Panel 1. Representative electron micrographs of egg. (A) Eggshell after hatching, bright field. (B) Region of eggshell showing spine. An EDS spot analysis, performed at the point marked on the spine (o_1_), is shown in Panel 2A. (C) Image of Formvar-carbon film. An EDS spot analysis (Panel 2B) was performed at the point indicated (o_1_). Bottom Panel (2) EDS spectra of spot analyses from Panel 1. (A) Spectra from region on spine (see spot on Panel 1B). (B) Spectra from region of the formvar-carbon supporting film (see spot marked on Panel 1C).

Energy dispersive spectroscopy (EDS) analysis of eggshells purified from faecal (Fig. 6, panels 1 and 2) and hepatic eggs (Fig. 7, panels 1 and 2) revealed consistent peaks for Fe. Similar EDS spectra for shells from eggs of faecal and hepatic origin indicate that the Fe in the hepatic eggshells is not derived by artifactual chelation of Fe from iron-rich hepatocytes during egg purification. Iron peaks were highest in the region of the fractured face of the eggshell. At these points, the shell material is contorted and folded, so that more eggshell is exposed to the electron beam. Fe was not detected on the carbon film in the vicinity of the eggshell (Fig. 6, Panel 2D, Fig. 7, Panel 2B), indicating that there is no iron background in either grids or microscope. Other elements present as above-background peaks include carbon, oxygen and copper (Cu). The low ratio of Cu-L to Cu-K peaks in EDS spectra of eggshells is consistent with indirect scattering from a thick source and represents scattering from the copper grid bars. The Fe-L to Fe-K peaks showed equivalent dimensions, indicating X-ray generation from a thin sample, which in these samples represents the eggs.

## 4 Discussion

This paper reports the first attempt to analyse gene expression in schistosome tissues using mRNA obtained by laser microdissection microscopy. This method has been identified as a means to rapidly analyse expression profiles of individual tissues of metazoan parasites (Jones, Gobert et al., 2004; Jones, Randall et al., 2004), proof of which was demonstrated recently by Ranjit, Jones, Stenzel, Gasser, and Loukas (2006), who constructed cDNA libraries of intestinal-specific tissues of hookworms. Whereas nematodes possess a body cavity and their component tissues are separated from each other by a pseudocoelom, the platyhelminths lack any form of body cavity; the tissues and organs of which are held together in a solid interstitial matrix (Conn, 1993) and are not easily separated. Given the solid nature of schistosome tissues, it was necessary in this study to determine the tissue constituency of vitellogenic regions to assess the relative abundance of vitelline cells in the region and to gain some insight into their likely contribution to ferritin expression. Ultrastructural observations of the vitelline tissues and other associated tissues were as noted by others (Erasmus, Popiel, & Shaw, 1982). Our study showed that interstitial cells, including cells of the musculo-parenchymal system are indeed present and hence may prevent any pure samples of specific tissue being obtained by laser microdissection. The vast majority of cellular material in vitellogenic regions (84%) belongs to vitelline cells, a highly synthetic cell type. These data implicate the vitelline cells as the likely source of yolk ferritin.

Northern blot analysis of yolk and somal ferritin expression patterns in *S. mansoni* revealed that males preferentially expressed somal ferritin while females preferentially expressed yolk ferritin (Dietzel et al., 1992). Our real-time PCR analysis of gender-specific expression of ferritins by *S. japonicum* presented here revealed similar patterns of expression. The abundance of transcripts in females and, specifically, in vitelline cells possibly reflect the high turnover of vitelline cells, and the high requirements for Fe stores in these cells.

It was also demonstrated here that yolk ferritin is expressed in miracidia, suggesting that this ferritin isoform, while preferentially expressed in females, has ubiquitous expression patterns. Lower expression levels of yolk ferritins, and the generally low expression levels of somal ferritin detected in all populations may reflect lower requirements for stored Fe in other tissues, or the longevity of ferritins in cells. In the human lung fluke, *Paragonimus westermani*, a homologous yolk ferritin was detected, as expected, in vitelline cells, but also in the early embryo of eggs retained in the parasite uterus (Kim, Joo, Kang, Cho, & Hong, 2002). It cannot be determined whether the intra-ovular ferritin of *P. westermani* was synthesised *de novo* by the larva, or scavenged from its uterine environment. Our observations of ferritin transcripts, suggest that for *S. japonicum*, at least, ferritins are synthesised by the embryo. Thus, it may be postulated, that the larva scavenges Fe from its intra-ovular environment, but not adult ferritin.

The role of Fe in function of the schistosome embryo, and hatched larva, is unclear. One hypothesis, mentioned earlier, was that of protection of the larva from bacterial attack (Schüssler et al., 1995). It is now thought, however, that the availability of Fe in snails may be an important regulatory factor in the host–parasite balance in the snail intermediate host (Lockyer, Spinks, Noble, Rollinson, & Jones, 2007). An alternative hypothesis, therefore, is that ferritin is produced in schistosome larvae to equip the schistosome larvae with Fe-stores for host interactions in its snail host.

Schistosome eggs hatch by a complex process that appears to require osmotic pressure within the egg, endogenous proteases and other larval activities (Xu & Dresden, 1990). For *S. japonicum*, the vitelline membrane and enclosed miracidium emerge entirely during hatching so that only shell remains. The eggshell is a dense polymerised protein matrix, with extensive topographical variation but little structural variation, as observed by electron microscopy (Cao, Wang, & Long, 1982; Neill, Smith, Doughty, & Kemp, 1988). We present in this study Fe detected in above-background levels in EDS and ICP-MS analyses of eggshells in both liver- and faeces-derived eggs.

Digenean trematodes synthesise eggshells from among a range of precursors proteins belonging to three families of tyrosine-rich proteins, the P14, P19 and P48 proteins (Ebersberger et al., 2005). In members of all three protein families, tyrosine residues are frequently flanked by glycine on the carboxy side (Waite & Rice-Ficht, 1992). The eggshell is formed by enzymatic oxidation of the tyrosine residues to dihydroxyphenylalanine (DOPA)-quinones, which then bind lysine residues of adjacent proteins (Cordingley, 1987; Eshete & LoVerde, 1993; Smyth & Clegg, 1959) to form a rigid structure that is resistant to enzymatic hydrolysis during passage from the host. Recently, it has been demonstrated that schistosome tyrosinases are critical in the cross-linking chemistry of the eggshells of *S. mansoni* (Fitzpatrick et al., 2007). The schistosome tyrosinases belong to a family of copper-dependant glycoenzymes. We therefore tested eggshell for Cu content to determine whether this element was present. Copper was found in trace amounts in the egg shell. This low abundance of the element may reflect the low abundance of the enzyme that remains entrapped in the shell.

Quinone-tanned proteins are produced by a range of organisms, particularly in those for which a strong, robust structure is required. The byssal threads of *Mytilus* sp., for example, are macroscopic protein polymers, core proteins of which are enriched in DOPA. Iron concentrations in byssal thread core proteins correlate with that of DOPA residues, suggesting that the interplay between oxidised products of tyrosine and metals are extremely important in protein polymerization (Sun & Waite, 2005). The presence of Fe as part of the quinone-tanned eggshell of schistosomes strongly implicates Fe in the chemistry of quinone tanning of the shells. If so, a primary role for vitelline Fe-stores is likely to be in choriogenesis. Furthermore, the yolk platelets, one of two major secretory components of vitelline cells and site of ferritin stores in vitelline cells (Schüssler et al., 1995), are likely contributors to eggshells and not to yolk.

In this study, we have shown that yolk ferritin is likely transcribed by vitelline cells and Fe, a storage component of ferritin, is incorporated into eggshells. Based on these studies, the models for eggshell formation in schistosomes and other platyhelminths (Colhoun, Fairweather, & Brennan, 1998; Wells & Cordingley, 1991, 1992) need to be revised to incorporate possible role and fate of vitelline stores of iron. Vitelline cells store and release eggshell precursors, as well as enzymes essential for choriogenesis (Wells & Cordingley, 1991). There are different vesicle compartments in the vitelline cells and each possibly contains the mix of precursors, enzymes and, we contend, Fe for shell formation. All components are released in the ootype of the female system, presumably under influence of the Mehlis gland (Colhoun et al., 1998). Quinone tanning efficiency is enhanced in the presence of Fe (Yamamoto et al., 1990), and an abundance of secreted Fe derived from vitelline cells may improve the efficiency of shell protein polymerization.

If this model holds, there are major implications for our understanding of the biology of platyhelminth vitelline cells. So far, the cells are reputed to secrete multiple proteins (Colhoun et al., 1998). If, as appears likely, there are multiple concurrent biosynthetic pathways occurring in these cells, then the vitelline cell of platyhelminths may prove to be truly complex cells. The full interplay between vitelline cells and ovum remain to be elucidated.

Severe consequences of human schistosomiasis arise because of the secretory activity of entrapped eggs in tissues (Ross et al., 2002). Although new efficacious vaccine targets against schistosomiasis have been identified (Tran et al., 2006) these do not produce sterilizing immunity and strategies to combat the severe pathological effects of disease are still necessary. In view of this, targeted studies of eggshell formation and possible degradation may lead to new strategies to minimise disease in schistosomiasis.

## Figures and Tables

**Table 1:** List of primers used for real time PCR and RT-PCR

Gene	Accession	Primer pair sequence (5′–3′)	
		Forward	Reverse
*S. japonicum* ferritin 1 (for real time PCR)	AF040385	gagcgacaacatgcgataaa	gtgtgtatcccgatgcctct
*S. japonicum* ferritin 2 (for real time PCR)	AY814600	ggatgcaatgaatacagctttg	ccaagtccttttccaacacg
*S. japonicum* ferritin 1 (for RT-PCR)	AF040385	atgtcgttgtgccgacaaaactaccat	ttaatcactttctccgtgtaaggtttc
*S. japonicum* ferritin 2 (for real time PCR)	AY814600	ccaaagaatgtgaagatgcta	tccaagtccttttccaacacg
*S. japonicum* TPI	U57557	aattgctgcacctttcgttta	ttttaccagattcacgctctga

**Table 2:** Elemental analysis of iron and copper content of schistosome egg components and host liver by ICP-MS

Description	Sample weight (mg)	Cu (mg/kg)	Fe (mg/kg)
Liver	145	3	159
Liver + eggs	190	2	270
Intact eggs	73	16	136
Eggshell only	97	8	121

## References

[bib1] Ashton P.D., Harrop R., Shah B., Wilson R.A. (2001). The schistosome egg: Development and secretions. Parasitology.

[bib2] Bartley P.B., Ramm G.A., Jones M.K., Ruddell R.G., Li Y., McManus D.P. (2006). A contributory role for activated hepatic stellate cells in the dynamics of *Schistosoma japonicum* egg-induced fibrosis. International Journal for Parasitology.

[bib3] Cao H.M., Wang Y.F., Long S. (1982). A study of ultrastructure of egg shell of *Schistosoma japonicum*. I. Transmission electron microscopic observation of *S. japonicum* egg. Annales de Parasitologie Humaine et Comparee.

[bib4] Clemens L.E., Basch P.F. (1989). *Schistosoma mansoni*: Effect of transferrin and growth factors on development of schistosomula *in vitro*. Journal of Parasitology.

[bib5] Colhoun L.M., Fairweather I., Brennan G.P. (1998). Observations on the mechanism of eggshell formation in the liver fluke, *Fasciola hepatica*. Parasitology.

[bib6] Conn D.B. (1993). The biology of flatworms (Platyhelminthes)—parenchyma cells and extracellular matrices. Transactions of the American Microscopical Society.

[bib7] Cordingley J.S. (1987). Trematode eggshells: Novel protein biopolymers. Parasitology Today.

[bib8] Dalton J.P., Day S.R., Drew A.C., Brindley P.J. (1997). A method for the isolation of schistosome eggs and miracidia free of contaminating host tissues. Parasitology.

[bib9] Dietzel J., Hirzmann J., Preis D., Symmons P., Kunz W. (1992). Ferritins of *Schistosoma mansoni*: Sequence comparison and expression in female and male worms. Molecular and Biochemical Parasitology.

[bib10] Ebersberger I., Knobloch J., Kunz W. (2005). Cracks in the shell—zooming in on eggshell formation in the human parasite *Schistosoma mansoni*. Development Genes and Evolution.

[bib11] Erasmus D.A., Popiel I., Shaw J.R. (1982). A comparative study of the vitelline cell in *Schistosoma mansoni*, *S. haematobium*, *S. japonicum* and *S. mattheei*. Parasitology.

[bib12] Eshete F., LoVerde P.T. (1993). Characteristics of phenol oxidase of *Schistosoma mansoni* and its functional implications in eggshell synthesis. Journal of Parasitology.

[bib13] Fitzpatrick J.M., Hirai Y., Hirai H., Hoffmann K.F. (2007). Schistosome egg production is dependent upon the activities of two developmentally regulated tyrosinases. FASEB Journal.

[bib14] Graca-Souza A.V., Maya-Monteiro C., Paiva-Silva G.O., Braz G.R., Paes M.C., Sorgine M.H. (2006). Adaptations against heme toxicity in blood-feeding arthropods. Insect Biochemistry and Molecular Biology.

[bib15] Griffiths G. (1993). Fine structure immunocytochemistry.

[bib16] Hoffmann K.F., Johnston D.A., Dunne D.W. (2002). Identification of *Schistosoma mansoni* gender-associated gene transcripts by cDNA microarray profiling. Genome Biology.

[bib17] Jones M.K., Gobert G.N., Zhang L., Sunderland P., McManus D.P. (2004). The cytoskeleton and motor proteins of human schistosomes and their roles in surface maintenance and host–parasite interactions. BioEssays.

[bib18] Jones M.K., Randall L., McManus D.P., Engwerda C. (2004). Laser microdissection microscopy in parasitology: Microscopes meet thermocyclers. Trends in Parasitology.

[bib19] Kim T.Y., Joo I.J., Kang S.Y., Cho S.Y., Hong S.J. (2002). *Paragonimus westermani*: Molecular cloning, expression, and characterization of a recombinant yolk ferritin. Experimental Parasitology.

[bib20] King C.H., Dickman K., Tisch D.J. (2005). Reassessment of the cost of chronic helmintic infection: A meta-analysis of disability-related outcomes in endemic schistosomiasis. Lancet.

[bib21] Kusel J.R. (1970). Studies on the structure and hatching of the eggs of *Schistosoma mansoni*. Parasitology.

[bib22] Lockyer A.E., Spinks J., Noble L.R., Rollinson D., Jones C.S. (2007). Identification of genes involved in interactions between *Biomphalaria glabrata* and *Schistosoma mansoni* by suppression subtractive hybridization. Molecular and Biochemical Parasitology.

[bib23] Moertel L., McManus D.P., Piva T.J., Young L., McInnes R.L., Gobert G.N. (2006). Oligonucleotide microarray analysis of strain- and gender-associated gene expression in the human blood fluke, *Schistosoma japonicum*. Molecular and Cellular Probes.

[bib24] Neill P.J., Smith J.H., Doughty B.L., Kemp M. (1988). The ultrastructure of the *Schistosoma mansoni* egg. American Journal of Topical Medicine and Hygeine.

[bib25] Oliveira G., Busek S., Correa-Oliveira R. (1998). Transcription levels of two actin genes (SmAct and SmAct2), cytochrome C oxidase subunit II (SmCOXII), triosephosphate isomerase (TPI), and a putative translation regulatory protein EIF-5 during the first seven days of in vitro development of *Schistosoma mansoni* schistosomula. Memórias do Instituto Oswaldo Cruz.

[bib26] Ranjit N., Jones M.K., Stenzel D.J., Gasser R.B., Loukas A. (2006). A survey of the intestinal transcriptomes of the hookworms, *Necator americanus* and *Ancylostoma caninum*, using tissues isolated by laser microdissection microscopy. International Journal for Parasitology.

[bib27] Rao A.U., Carta L.K., Lesuisse E., Hamza I. (2005). Lack of heme synthesis in a free-living eukaryote. Proceedings of the National Academy of Sciences of the United States of America.

[bib28] Ross A.G., Bartley P.B., Sleigh A.C., Olds G.R., Li Y., Williams G.M. (2002). Schistosomiasis. New England Journal of Medicine.

[bib29] Schüssler P., Potters E., Winnen R., Bottke W., Kunz W. (1995). An isoform of ferritin as a component of protein yolk platelets in *Schistosoma mansoni*. Molecular Reproduction and Development.

[bib30] Schüssler P., Potters E., Winnen R., Michel A., Bottke W., Kunz W. (1996). Ferritin mRNAs in *Schistosoma mansoni* do not have iron- responsive elements for post-transcriptional regulation. European Journal of Biochemistry.

[bib31] Smyth J.D., Clegg J.A. (1959). Egg-shell formation in trematodes and cestodes. Experimental Parasitology.

[bib32] Smyth D., Glanfield A., McManus D.P., Hacker E., Blair D., Anderson G.J. (2006). Novel divalent metal transporters (DMT1) for *Schistosoma mansoni* suggest a pathway for iron absorption in schistosomes. Journal of Biological Chemistry.

[bib33] Sun C., Waite J.H. (2005). Mapping chemical gradients within and along a fibrous structural tissue, mussel byssal threads. Journal of Biological Chemistry.

[bib34] Torrance J.D., Bothwell T.H. (1968). A simple technique for measuring storage iron concentrations in formalinised liver samples. South African Journal of Medical Sciences.

[bib35] Tran M.H., Pearson M.S., Bethony J.M., Smyth D.J., Jones M.K., Duke M. (2006). Tetraspanins on the surface of *Schistosoma mansoni* are protective antigens against schistosomiasis. Nature Medicine.

[bib36] Waite J.H., Rice-Ficht A.C. (1992). Eggshell precursor proteins of *Fasciola hepatica*. II. Microheterogeneity in vitelline protein B. Molecular and Biochemical Parasitology.

[bib37] Walcourt A., Loyevsky M., Lovejoy D.B., Gordeuk V.R., Richardson D.R. (2004). Novel aroylhydrazone and thiosemicarbazone iron chelators with anti-malarial activity against chloroquine-resistant and -sensitive parasites. International Journal of Biochemistry and Cell Biology.

[bib38] Wells K.E., Cordingley J.S. (1991). *Schistosoma mansoni*: Eggshell formation is regulated by pH and calcium. Experimental Parasitology.

[bib39] Wells K.E., Cordingley J.S. (1992). The cell and molecular biology of eggshell formation in *Schistosoma mansoni*. Results and Problems in Cell Differentiation.

[bib40] Wilson M.E., Britigan B.E. (1998). Iron acquisition by parasitic protozoa. Parasitology Today.

[bib41] WoldeMussie E., Bennett J.L. (1982). Plasma spectrometric analysis for Na, K, Ca, Mg, Fe, and Cu in *Schistosoma mansoni* and *S. japonicum*. Journal of Parasitology.

[bib42] Xu Y.Z., Dresden M.H. (1990). The hatching of schistosome eggs. Experimental Parasitology.

[bib43] Yamamoto H., Kuno S., Nagai A., Nishida A., Yamauchi S., Ikeda K. (1990). Insolubilizing and adhesive studies of water-soluble synthetic model proteins. International Journal of Biological Macromolecules.

